# Computational Design of Radical Recognition Assay with the Possible Application of Cyclopropyl Vinyl Sulfides as Tunable Sensors

**DOI:** 10.3390/ijms22147637

**Published:** 2021-07-16

**Authors:** Liliya T. Sahharova, Evgeniy G. Gordeev, Dmitry B. Eremin, Valentine P. Ananikov

**Affiliations:** Zelinsky Institute of Organic Chemistry, Russian Academy of Sciences, Leninsky Prospect 47, 119991 Moscow, Russia; liliya685@ioc.ac.ru (L.T.S.); gordeev_e@ioc.ac.ru (E.G.G.); erem@ioc.ac.ru (D.B.E.)

**Keywords:** radicals, molecular dynamics, recognition assay, sensors, radical clock, radical traps

## Abstract

The processes involving the capture of free radicals were explored by performing DFT molecular dynamics simulations and modeling of reaction energy profiles. We describe the idea of a radical recognition assay, where not only the presence of a radical but also the nature/reactivity of a radical may be assessed. The idea is to utilize a set of radical-sensitive molecules as tunable sensors, followed by insight into the studied radical species based on the observed reactivity/selectivity. We utilize this approach for selective recognition of common radicals—alkyl, phenyl, and iodine. By matching quantum chemical calculations with experimental data, we show that components of a system react differently with the studied radicals. Possible radical generation processes were studied involving model reactions under UV light and metal-catalyzed conditions.

## 1. Introduction

Reactions involving radicals play a key role in photonics, catalysis, life sciences, and medicine. Computational modeling of reactive radical species and computational design of new materials are areas of paramount importance since highly reactive radical molecules are difficult to isolate and characterize experimentally [1,2,3,4,5,6,7,8,9,10,11,12]. Recent decades have seen a renaissance in radical chemistry [13,14,15,16,17,18,19,20,21]. In practical applications, radical and photochemical reactions play a pivotal role in the synthesis of various organic compounds and drugs [22,23,24,25,26,27,28,29], functionalization of heterocyclic systems [30], photoredox catalysis [31,32,33], and materials science [34].

**Scheme 1:**
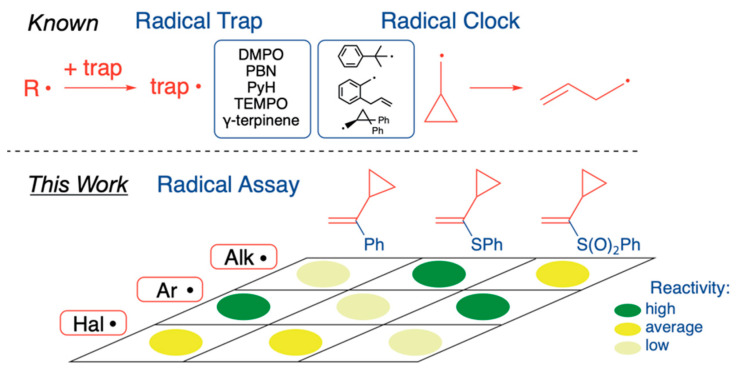
Different ways of recognition and trapping radicals with a general idea of a radical assay.

Radicals are highly reactive species that can interact with organic molecules. Often, due to the high reactivity of radicals, it is challenging to study the mechanism of the reaction and determine what type of radical is involved [35,36]. Thus, to study the mechanisms of radical reactions, researchers use various tools, such as radical traps (Scheme 1) [37]. The method of radical trapping is to react free radicals formed during the reaction with special acceptors, which are capable of binding free radicals and forming detectable species [38,39,40].

Another way to establish the radical nature of a reaction involves the use of a radical clock (Scheme 1) [41,42,43]. The “radical clock” tool indirectly studies the reaction proceeding through the formation of radical intermediates [44]. By virtue of radical traps or radical clocks, one can assume evident that of radicals present and time their behavior. 

However, at the same time, we often cannot confidently speak about the reaction mechanism and which radical is involved in the reaction.

In this work, we propose an approach that allows one to investigate the participation of certain radicals in different reactions. For the first time, we have added sulfur-functionalized groups to the vinyl cyclopropyl framework of a radical clock. The novelty of the present study is to propose the concept of a radical recognition assay and perform modeling and molecular dynamics (MD) simulations of a radical trapping process. The interaction of these compounds with Alk•, Ar•, or Hal• leads to the opening of the cyclopropane ring. However, the presence of a -S- or SO_2_- group at a double bond may influence the process. Thus, knowing how the selected compounds interact with radicals of various natures, we can further use the obtained output reactivity matrix as a “sensor” for certain radicals (Scheme 1).

As noted above, along with 1,1-cyclopropyl-phenylethylene, we have chosen corresponding sulfur derivatives as radical sensors. Our choice was determined by a combination of a cyclopropane ring and a sulfur atom at a double bond within one molecule. The cyclopropane ring readily undergoes opening reactions, including radical-initiated ones, and is often used as a radical clock [45]. Notably, a sulfur atom may be used as a radical stabilizer, and the introduction of this atom into a molecule can affect the course of reactions involving radicals [46,47,48].

In our work, Markovnikov-type vinyl sulfides were the primary focus since they provide a predetermined site of attack in radical reactions and are isostructural to the classical radical clock. Predicting the possible reaction space and obtaining the overall reactivity matrix are the key stages for developing a radical recognition assay.

## 2. Results

**Figure 1:**
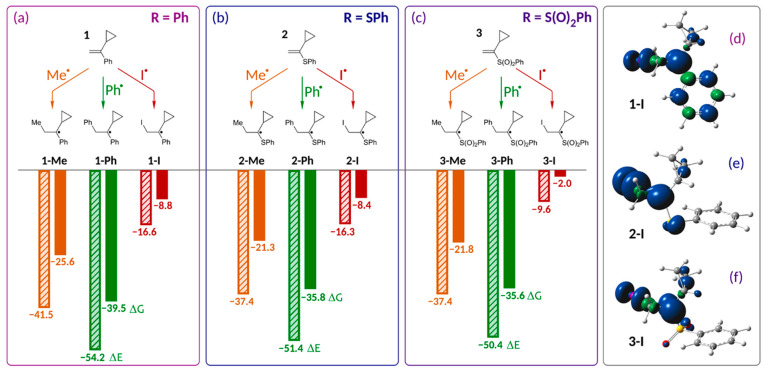
(**a**–**c**) The values of the reaction total energies ΔE (diagonal shading) and reaction free energies ΔG (solid fill, kcal/mol) for reactions of compounds **1**–**3** with different radicals. (**d**–**f**) Spatial distribution of spin density for products of interaction of alkenes **1**–**3** with iodine radical (Figures S3 and S4 for spin density analysis of other addition products); UPBE1PBE/6-311 + G(d,p) D3BJ level.

Thus, to explain the regioselectivity of the addition of radicals and different activities of the radical trapping molecules, quantum-chemical computational modeling of the reactions of compounds **1–3** with Alk•, Ar•, and I• (the radical capture stage, since it is rate-determining) was carried out at the UPBE1PBE/6-311+G(d,p) D3BJ level (Figure 1, see also Supplementary Materials). The addition of a radical to molecules **1**, **2**, and **3** involves the unsubstituted carbon atom of the double bond. In products **1-Ph**, **2-Ph**, and **3-Ph**, the C_Ph_-C interatomic distances are 1.517, 1.516, and 1.513 Å, respectively, and the C_Me_-C interatomic distances in the **1-Me**, **2-Me**, and **3-Me** products are 1.534, 1.535, and 1.536 Å, respectively (Figure S1). These values are in agreement with the stronger bonding of the Ph• radical. The addition of Alk• or Ph• results in the formation of a strong covalent bond; when this happens, the terminal carbon atom of the double bond of the substrate molecule acquires a tetrahedral configuration. The addition of I• more or less preserves the planar shape of the terminal carbon atom environment. For instance, the calculated C•-C-H angles in products **3-Me** and **3-I** are 108.4° and 113.5° for Alk• and I•, respectively (where C^•^-C is the former alkene double bond), which confirms the weaker bonding of I•. Indeed, the highest binding energies are typical for alkyl and aryl radicals, whereas the exergonic effect of the iodine radical addition is much smaller (Figure 1), which fits well with the high activity of carbon-centered radicals observed in the experiments.

**Figure 2:**
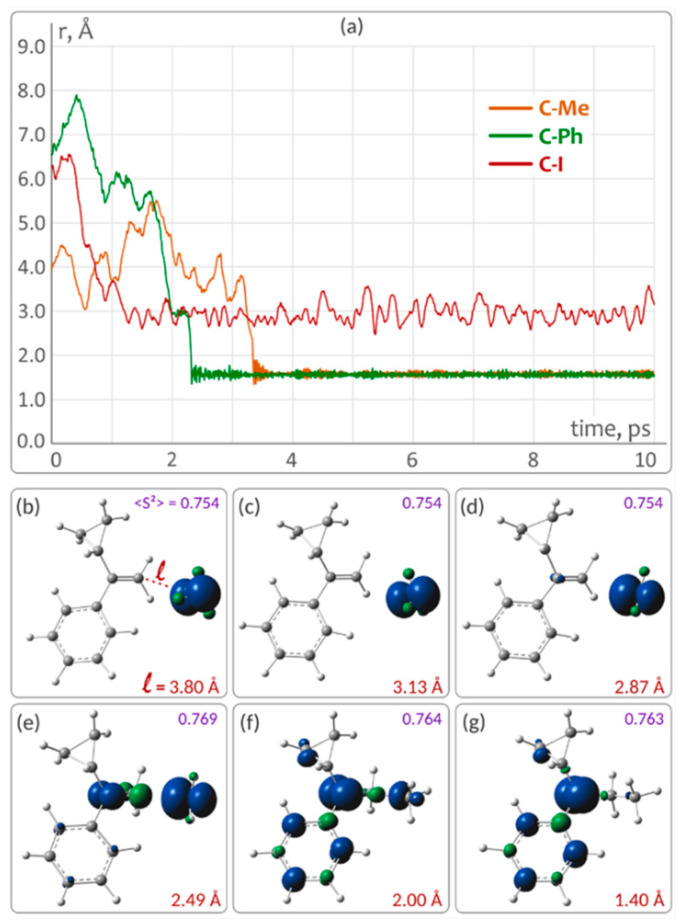
(**a**) Dynamics of the C-C_Me_, C-C_Ph_, and C-I interatomic distances for the interaction processes of the methyl radical, phenyl radical, and iodine radical with compound **1**; (**b**–**g**) some MD-trajectory frames of the spin density dynamics for the addition of Alk• radical to compound **1** (the C-C interatomic distance between radical carbon atom and terminal carbon atom of alkene are shown in red for each structure; the values of the <S^2^> are shown in purple for each structure).

To analyze the reactivity of different radicals in the addition reaction to the considered molecules, molecular systems consisting of alkene **1** and each of the radicals (Alk•, Ar• or I•) were modeled by DFT molecular dynamics (see Figure 2a). During the MD simulation, the radical may bond to any of the carbon atoms of the double bond so that the regioselectivity of the addition reaction can be analyzed.

For Alk•, 11 trajectories lead to the formation of an addition product at the double bond, while 4 trajectories lead to the formation of a methane molecule (Figures S7–S9). During the addition of the alkyl radical to the double bond, the unpaired electron is completely localized on the Me• carbon atom until it approaches the terminal carbon atom of the alkene to a distance of approximately 3 Å (Figure 2b,c).

Further approach to 2.5 Å leads to polarization of the orbital, on which the unpaired electron is localized and partial electron transfer to the atoms of the double bond occurs (Figure 2d,e). At a distance of 2 Å, the unpaired electron is more localized on the double bond atoms (Figure 2f), and full localization of the electron on the tertiary carbon atom occurs during the final formation of the covalent C-C bond (Figure 2g). Throughout the trajectory of addition of the Me radical, the values of <S^2^> do not significantly exceed the exact value of <S^2^> = 0.75; that is, spin contamination is insignificant in this case.

In the case of Ar•, the formation of the addition product at the double bond occurs in 7 trajectories out of 15 (Figures S7 and S10). In five cases, the formation of a benzene molecule occurs: in one case, the formation of a benzene molecule occurs as a result of the transfer of a hydrogen atom from the secondary carbon atom of the cyclopropyl substituent (Figure S11), and in other cases, the formation of a benzene molecule is realized due to the transfer of a hydrogen atom from the side of a tertiary carbon atom, as in the case of the formation of a methane molecule (Figure S12). In three trajectories, Ar• is bonded to the ortho-position of the phenyl substituent of the alkene molecule (Figure S13). Thus, Ar• represents a more active species than Alk•, which may explain the lower amount of the final product in the case of Ar•. The activity of Ar• is so high that it begins to interact with an organic molecule “without waiting” for a collision with a double bond and interacts with those groups that are closest during a given segment of the trajectory. In addition, the effective collision of Ar• with the double bond is realized in fewer cases due to the larger spatial volume of this particle. That is, high activity combined with a large volume can explain the lower yield of the final product for Ar• in some cases (experimental data are discussed below).

The activity of I• is insufficient to initiate the dissociation of the C-H bond or to bond with a phenyl substituent (since the addition to the phenyl substituent is accompanied by a breaking of its aromaticity). I• is relatively quickly coordinated with the terminal carbon atom of the double bond and forms an addition product **1-I** in all 15 trajectories without exceptions (Figure S14). Figure 2a shows fragments of MD trajectories with a length of 10 ps, on which a radical particle binds to a terminal carbon atom. These trajectory fragments illustrate the dynamics of changes in the C-C_Ph_, C-C_Me_, and C-I interatomic distances for each of the radicals and are superimposed on the time scale solely for ease of comparison. As seen from the figure, the formation of C-C bonds occurs abruptly, and further fluctuations in the C-C interatomic distance are insignificant. The process of C-I bond formation is longer in time and occurs gradually, while the C-I interatomic distance continues to vary within wide limits; that is, the C-I bond retains its high lability. For comparison, MD modeling of the addition of an iodine radical to molecule **1** by the UPBE/6-31G(d) D3 method was performed (Figure S15), and this method also confirms the high lability of the C-I bond (the amplitude of the change in the C-I interatomic distance is approximately 0.5 Å).

However, the radical binding efficiency is defined not only by the activity of radical particles but also by the nature of trapping molecules. The considered vinyl sulfides and vinyl sulfones significantly differ in their radical binding capacities.

For instance, the most significant exergonic effect is observed for the addition of radical particles to substrate **1**, which lacks sulfur atoms in its structure (Figure 1a). The phenyl group is actively involved in unpaired electron delocalization in the reaction product, as indicated by the spin density distribution (Figure 1d, Figures S3 and S4).

Introducing a sulfur atom into the trapping molecule completely blocks delocalization of the unpaired electron onto the phenyl substituent and results in greater localization of the unpaired electron on the carbon atom of the double bond (Figure 1e,f, Figures S3 and S4). The localization of the unpaired electron on the SO_2_ group in compounds **3-Me**, **3-Ph**, and **3-I** is much less than that on the sulfur atom in compounds **2-Me**, **2-Ph**, and **2-I**.

Thus, the more pronounced exergonic effect of the reaction with compound **1** can be explained by extra stabilization of the reaction product due to the expanded delocalization of the unpaired electron with the involvement of the phenyl group. Compound **3** shows the weakest interaction with I•, which perfectly fits the experimental data (Figure 1c and discussion below).

Thus, a significant change in the radical trap efficiency with the introduction of heteroatoms into the molecule is quite comprehensible from the standpoint of quantum chemistry. Compounds **1** and **2** can trap various radicals, whereas compound **3**, with its SO_2_R moiety, selectively interacts with the most active radical particles.

**Scheme 2:**
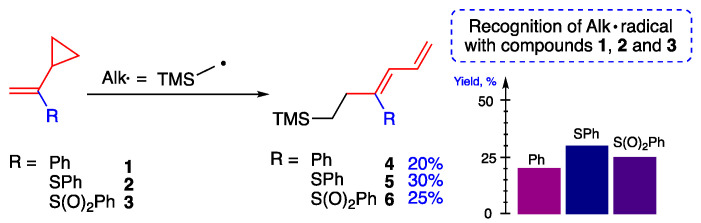
Cyclopropane ring-opening reactions of compounds **1**, **2**, and **3** with Alk•.

In the next stage, we synthesized compounds **1**, **2**, and **3** and checked their activity in the presence of Alk•, Ar•, or I• for evaluation of the design data. We examined whether the selected compounds participate in radical-clock-type transformations. The interaction with Alk• was studied first; the model reaction involved the formation of Alk• in the presence of Pd(OAc)_2_ (Scheme 2) [49].

Compounds **1**, **2**, and **3** showed comparable reactivities towards Alk• in this reaction. The most active of the three compounds was **2**, with a 30% yield of the ring-opening product.

**Scheme 3:**
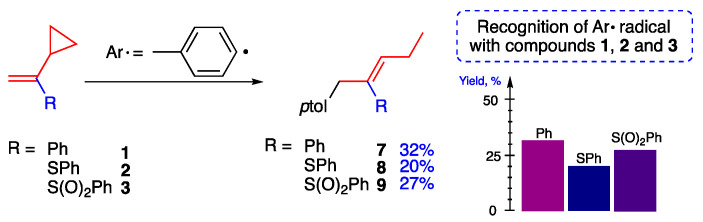
Cyclopropane ring-opening reactions of **1**, **2**, and **3** with Ar radical.

In the next step, we studied the same compounds in the reaction with Ar• by using para-iodotoluene as its source (Scheme 3) [50]. As in the previous experiment with Alk• radical, compounds **1**, **2**, and **3** reacted by ring opening, with comparable yields of the products. However, the reaction of **2** towards Ar• gave a lower yield and was significantly lower than the reaction with Alk• in the previous set.

**Scheme 4:**
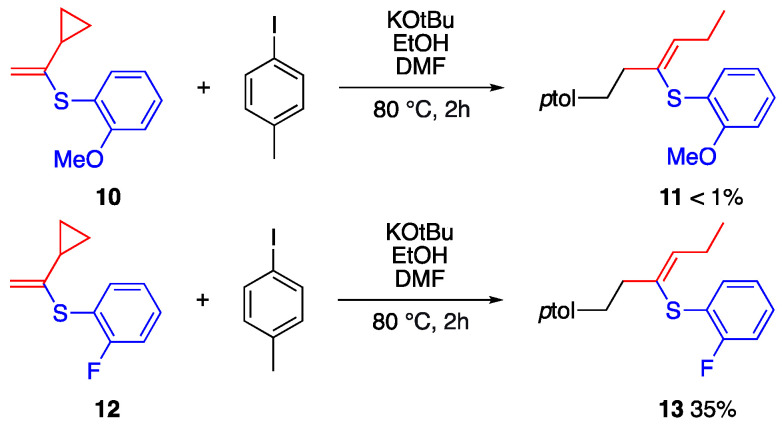
Cyclopropane ring-opening reactions of cyclopropylstyrenes **10** and **12** with Ar radical.

We tested the possibility of tuning the yields of ring opening by putting electron-withdrawing or electron-donating substituents onto the aromatic ring of 1,1-cyclopropyl-phenylethylene (Scheme 4). The same reaction was conducted using synthesized compounds with electron-donating OMe group **10** and with electron-withdrawing F group **12** (Scheme 4). The presence of the electron-donating substituent suppressed ring opening, probably by destabilizing the radical. Conversely, ring-opening enhancement was observed for **12**, as the electron-withdrawing group stabilized the radical.

**Scheme 5:**
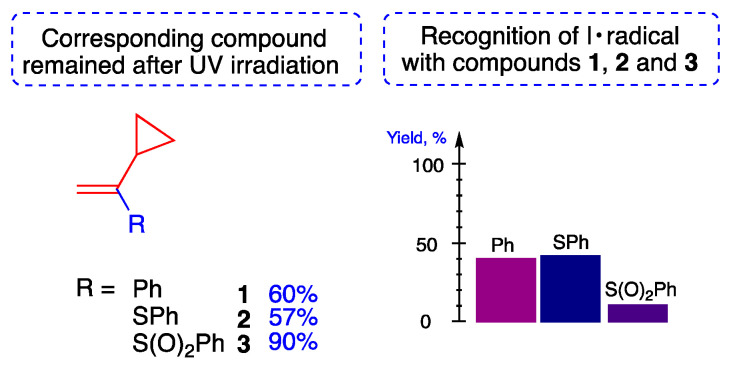
Stability of Compounds 1, 2, and 3 under UV irradiation with N-iodosuccinimide.

Finally, we tested the reactivity of compounds **1**, **2**, and **3** towards I• radicals. In this series of experiments, we treated each of the compounds with N-iodosuccinimide under UV irradiation for 30 min. Compound **3** turned out to be the most stable to I•, with only 10% decomposition in the experiment (Scheme 5).

## 3. Conclusions

To summarize, the proposed radical recognition assay interacts with radicals of various natures; the radical-induced transformations proceed via cyclopropane ring opening and can be easily described. Depending on the nature of the radicals, we observed differential reactivity of the obtained compounds towards active species. By matching experimental and computational data, we show that introducing a sulfide connection next to cyclopropyl increases selectivity towards radical clock reactions with alkyl radicals while decreasing the yield of radical-clock-type products with aryl radicals. As indicated by MD simulations, lower selectivity towards the formation of radical clock products may be expected in the reaction with the Ph radical. In contrast, introducing a sulfone group at the same position leads to a decrease in the reactivity of the molecule towards iodine radicals while retaining the reactivity towards carbon-centered radicals. Due to the differential reactivity of the products towards radicals, the developed system may be used as a sensor to determine the nature of radicals involved in chemical reactions.

Today, there are different ways to recognize radicals. However, available methods typically answer the following question: is there a radical in the system or not? In our work, we tried to answer the question: which radical takes part in the reaction? Due to the ability of synthesized molecules to interact with some radicals better than with others, we proposed the idea of a radical trapping assay. A system of interest is evaluated towards various radical traps (not a single radical trap) of different natures. A combined outcome is analyzed and can be used to distinguish reaction radicals.

Of course, here, we describe only the general idea of a radical recognition assay together with preliminary experiements. Further studies are required to assess its practical potential. More precise generation of pure radicals would be required to calibrate and test the radical assay, while detailed studies on reactivity are needed for further development. Here, we propose the idea of using radical assays and anticipate future studies on the applicability of various radical trapping assays.

## Data Availability

Data are contained in the article and Supplementary Materials.

## Supplementary Materials

Supplementary materials can be found at https://www.mdpi.com/article/10.3390/ijms22147637/s1.
