# Supplementary material for: Computational Design of Radical Recognition Assay with the Possible Application of Cyclopropyl Vinyl Sulfides as Tunable Sensors
Source: Int J Mol Sci. 2021 Jul 16;22(14):7637. doi: 10.3390/ijms22147637 (PMC8306039; doi:10.3390/ijms22147637)
Supplement: Supplementary file 1 [file ijms-22-07637-s001.zip › ijms-1268870-supplementary.pdf]

Supporting Information

Computational Design of Radical Recognition Assay with the Possible Application of Cyclopropyl Vinyl Sulfides as Tunable Sensors

Liliya T. Sahharova, Evgeniy G. Gordeev, Dmitry B. Eremin, Valentine P. Ananikov*

Zelinsky Institute of Organic Chemistry, Russian Academy of Sciences, Leninsky Prospect 47, Moscow, 119991, Russia

* e-mail: val@ioc.ac.ru

1. Materials and Methods S2

2. Computational details.. S2

3. Computational study.. S5

4. Experimental study.. S18

References S22

1. Materials and Methods

General procedures

Rreactions were performed in screw capped glass tubes with magnetic stir bars. Ragents were purchased from commercial sources and checked by ^{1}H and ^{13}C NMR spectroscopy prior to use. The yields were evaluated by ^{1}H NMR spectra. $CDCl_3$, $(CD_3)_2CO$, and $(CD_3)_2SO$ for NMR spectroscopy were obtained from Deutero GmbH and used as received. LC-MS-grade solvents for ESI-MS experiments were obtained from Merck and used fresh as purchased. All samples for the ESI-MS experiments were prepared in 1.8 mL Agilent screw top glass vials.

- *NMR Experiments*

NMR measurements were performed using a Bruker DRX500 spectrometer equipped with a 5-mm BBO probe head operating at 500.1, 125.8, and 470.5 MHz for ^{1}H and ^{13}C, respectively, or using a Bruker AVANCE 400 spectrometer at 400.1, 100.1, and 376.4 MHz for ^{1}H, ^{13}C, and ^{19}F, respectively, or using a Bruker Fourier HD300 spectrometer at 300.1, 75.5 MHz for ^{1}H and ^{13}C, respectively, in $CDCl_3$, $(CD_3)_2CO$, $(CD_3)_2SO$. The spectra of reaction products were acquired immediately after the reactions and processed using the TopSpin 4.0 software package. The ^{1}H and ^{13}C chemical shifts were referenced to internal standards provided by the solvent.

- *ESI-MS Measurements*

High-resolution mass spectra were recorded on a Bruker maXis Q-TOF instrument equipped with an electrospray ionization (ESI) ion source. The measurements were performed in positive (+) MS ion mode (HV capillary: 4500 V; spray shield offset: −500 V) with a scan range of m/z 50 − 3000. External calibration of the mass spectrometer was performed using a freshly prepared sodium formate calibrant solution. Direct syringe injection was used for all of the analyzed solutions in MeCN (flow rate: 3 μL min^{-1}). Nitrogen was used as the nebulizer gas (0.4 bar) and dry gas (4.0 L min^{-1}, 180 °C). All recorded spectra were processed using the Bruker Data Analysis 4.0 software package.

2. Computational details

Analysis of the reaction energies for radical addition processes

All molecular structures were optimized by the UPBE1PBE[1] method with a 6-311+G(d,p)[2] basis set (for iodine radicals, the def2TZVP basis set[3] was applied). For an accurate description of

the dispersion interaction, D3BJ empirical corrections were used.[4] For each structure, the vibrational spectrum was calculated. All calculations were performed by Gaussian 16 software.[5] Optimized molecular structures were visualized by the CYLView program[6], and spin density distribution was visualized by GaussView 6.0 software[7] with a medium grid.

Molecular dynamics of the radical addition reactions

To study the regioselectivity of the addition of radical particles to alkene **1** molecule, we simulated these processes by DFT molecular dynamics. For each radical, a simulation of a molecular system consisting of one radical particle and one alkene molecule was performed. Each molecular system before MD simulation was fully optimized, resulting in the formation of an intermolecular complex containing a radical particle and alkene binding by weak intermolecular interactions. That is, in the molecular structure used as the initial structure for MD simulation, the radical during the simulation could bind to both one and the other carbon atom of the multiple bond of the trap molecule. Since the modeled system consists of a small number of atoms and is not surrounded by solvent molecules, a separate stage of MD equilibration was not carried out and the whole trajectory was used for the analysis. MD modeling was performed using the UPBE D3 method and the STO-3G basis set[8] for all elements except iodine. The LanL2DZdp basic set[9] was used for iodine. The simulated molecular system was placed in a spherical potential (nanoreactor) that holds the fragments of the system in a confined space. The radius of the nanoreactor was 4.5 Å for systems containing a methyl radical and an iodine radical and 5.0 Å for a system containing a phenyl radical. MD simulation was performed in the NVT ensemble at 400 K (Langevin thermostat). MD timestep was equal to 0.5 fs. In all cases, the total trajectory length was set to 200 ps. But in each specific case, the actual length of the trajectory was different, since the modeling was carried out before the onset of a chemical event: the addition of a radical to a double bond, the formation of a molecule of methane or benzene, etc. In all cases, the chemical interaction of the radical with the alkene molecule occurred earlier than 200 ps, since the molecules were placed in a nanoreactor, which increases the probability of their interaction. The resulting product of the interaction of the radical with the alkene is a stable compound and its decomposition into the initial fragments at a given temperature is unlikely; therefore, after the formation of the addition product, MD simulation was terminated.

For all systems, a polarizable continuum model PCM was used. Toluene, DMF and DMSO were the solvents applied in the experimental work for reactions with Me-, Ph- and I-radicals correspondingly.

Comparison of the UPBE D3/STO-3G&LanL2DZdp(I) and UPBE1PBE D3BJ/6-311+G(d,p)&def2TZVP(I) methods (Figures S1 and S2) shows that, despite its rigidity, the

minimal STO-3G basis set correctly describes the geometry of the starting alkenes and radical intermediates, as well as the spin density distribution in the latter. In particular, the deviation of the C-C and C-S interatomic distances between these two methods is 0.02-0.08 Å. The maximum discrepancy between the two methods is observed in the case of the C-I interatomic distance, which is 2.25-2.50 Å for the UPBE1PBE D3BJ/6-311+G(d,p)&def2TZVP(I) method and 2.54-2.71 for the UPBE D3/STO-3G&LanL2DZdp(I). However, it should be noted that the relative changes in the interatomic distance C-I during the transition from one intermediate to another are in good agreement for these two levels of the theory. For the method with a more extended basis set, the C-I interatomic distances are 2.27, 2.50, and 2.25 Å for intermediates **1-I**, **2-I**, and **3-I** correspondingly, and for the minimum basis set, these interatomic distances are 2.55, 2.71, and 2.54 Å. The distribution of spin density in the products of radical particle addition is also in good agreement between the two methods (Figures S3 and S5): in the absence of a sulfur atom, the unpaired electron is delocalized to the phenyl group at the double bond, while the presence of a sulfur atom between the double bond and the phenyl substituent blocks such delocalization.

The differences in the energies of radical addition reactions calculated by these methods are quite significant (Figure S6); nevertheless, the method of a lower level UPBE D3/STO-3G&LanL2DZdp(I) results in a correct changes in the activity of radicals: in all cases, the most active is phenyl radical, and the least active is the iodine radical, and in this matter the methods are in good agreement with each other.

Thus, UPBE D3/STO-3G&LanL2DZdp(I) method can be used to perform MD modeling of these radical reactions as a computationally inexpensive DFT method. The study of regioselectivity requires obtaining statistical data, that is, calculating a set of MD trajectories to analyze the distribution of various reaction products. The use of more accurate and expensive methods in this case is too time consuming, and the use of semiempirical methods can lead to incorrect results, since the parametrization of semiempirical methods for radical particles may not be complete enough.

All MD calculations were performed using the TeraChem 1.93P software package.[10] Visualization and analysis of the obtained trajectories were performed using the VMD program.[11]

3. Computational study

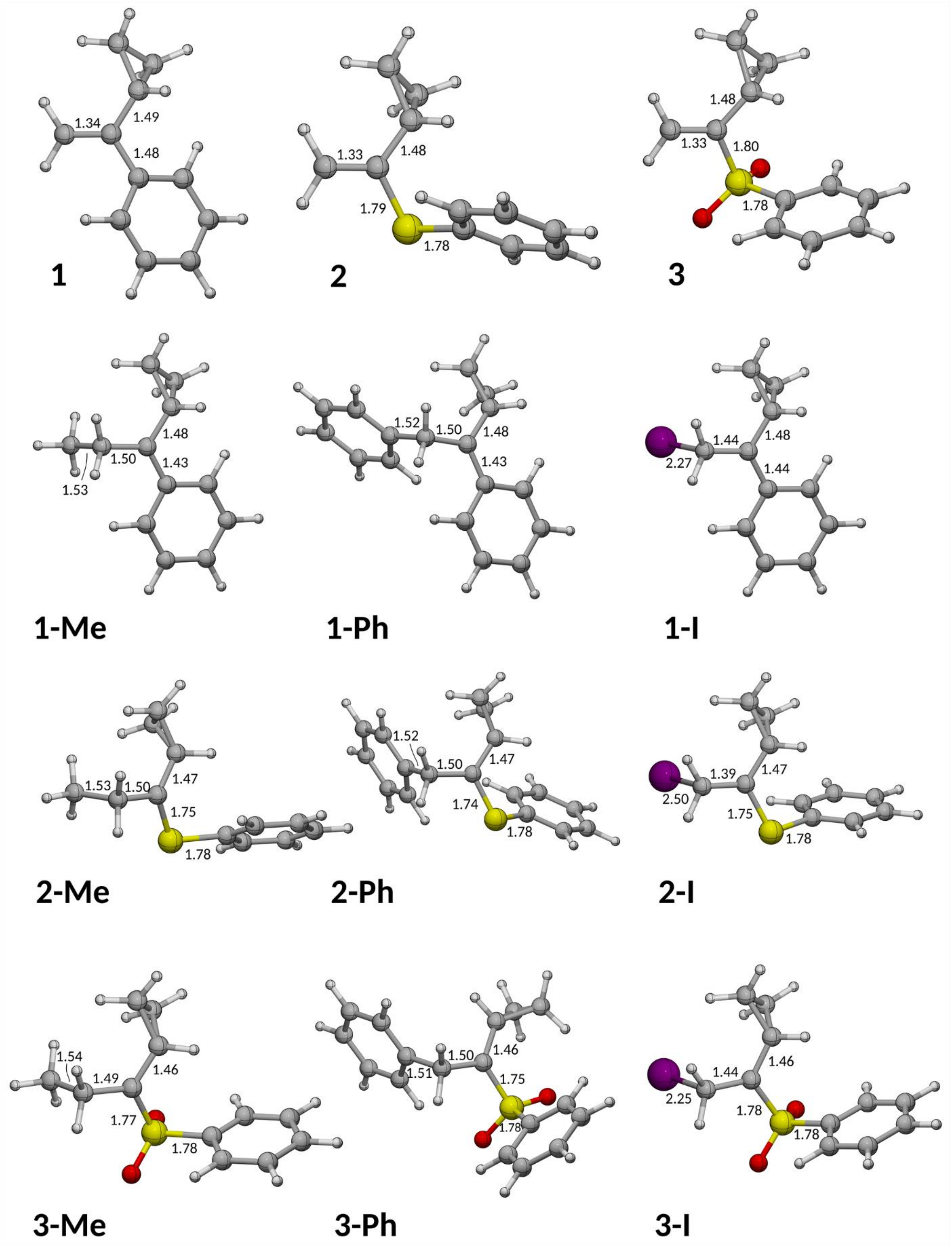

Figure S1. Optimized molecular structures of initial radical clock molecules (**1**-**3**), intermediate products of Me-radical addition (**1-Me** - **3-Me**), intermediate products of Ph-radical addition (**1-Ph** - **3-Ph**) and intermediate products of I-radical addition (**1-I** - **3-I**). Interatomic distances are shown in angstroms, UPBE1PBE/6-311+G(d,p) D3BJ.

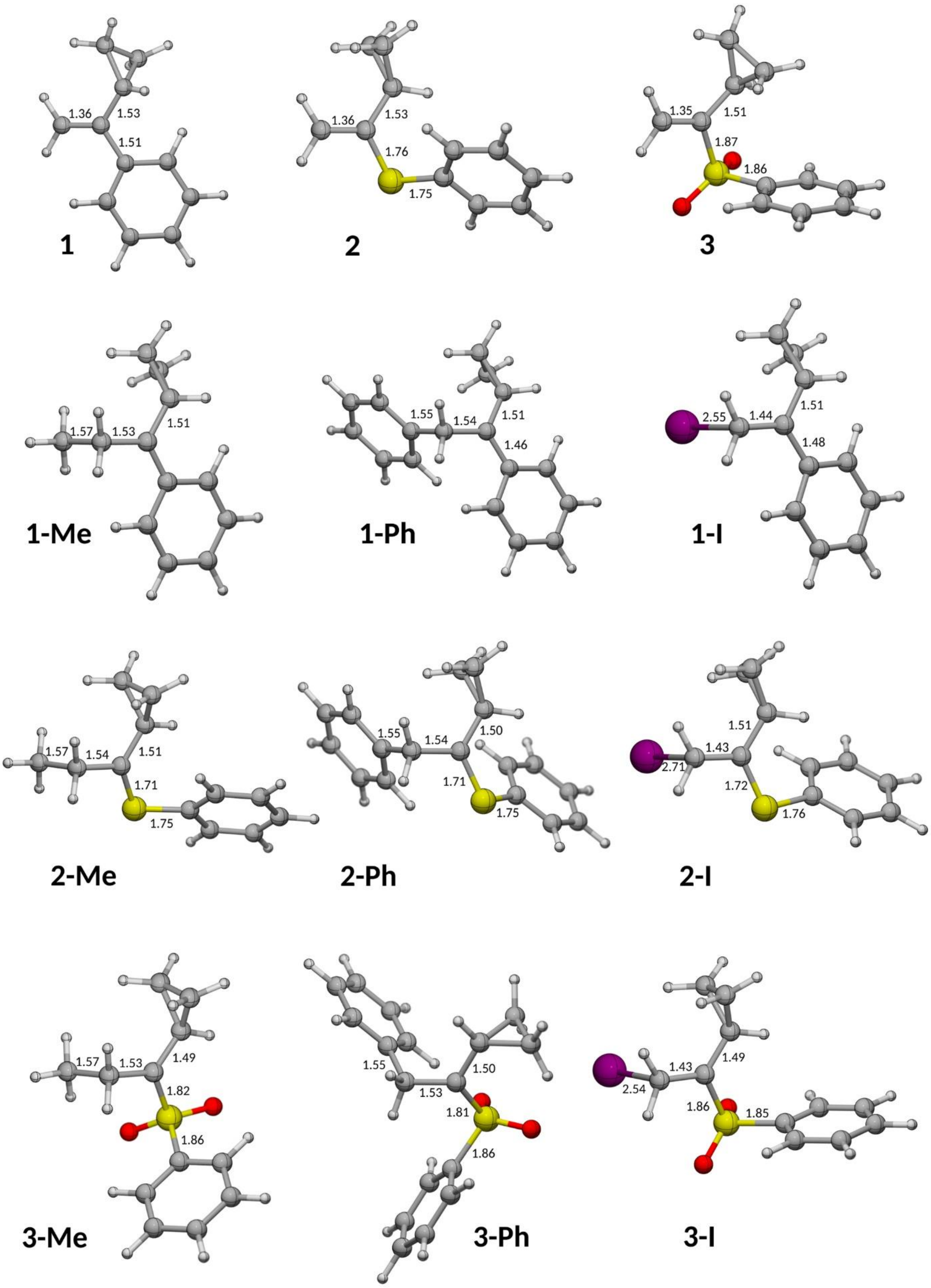

Figure S2. Optimized molecular structures of initial radical clock molecules (**1**-**3**), intermediate products of Me-radical addition (**1-Me** - **3-Me**), intermediate products of Ph-radical addition (**1-Ph** - **3-Ph**) and intermediate products of I-radical addition (**1-I** - **3-I**). Interatomic distances are shown in angstroms, UPBE/STO-3G D3 (LanL2DZdp for I).

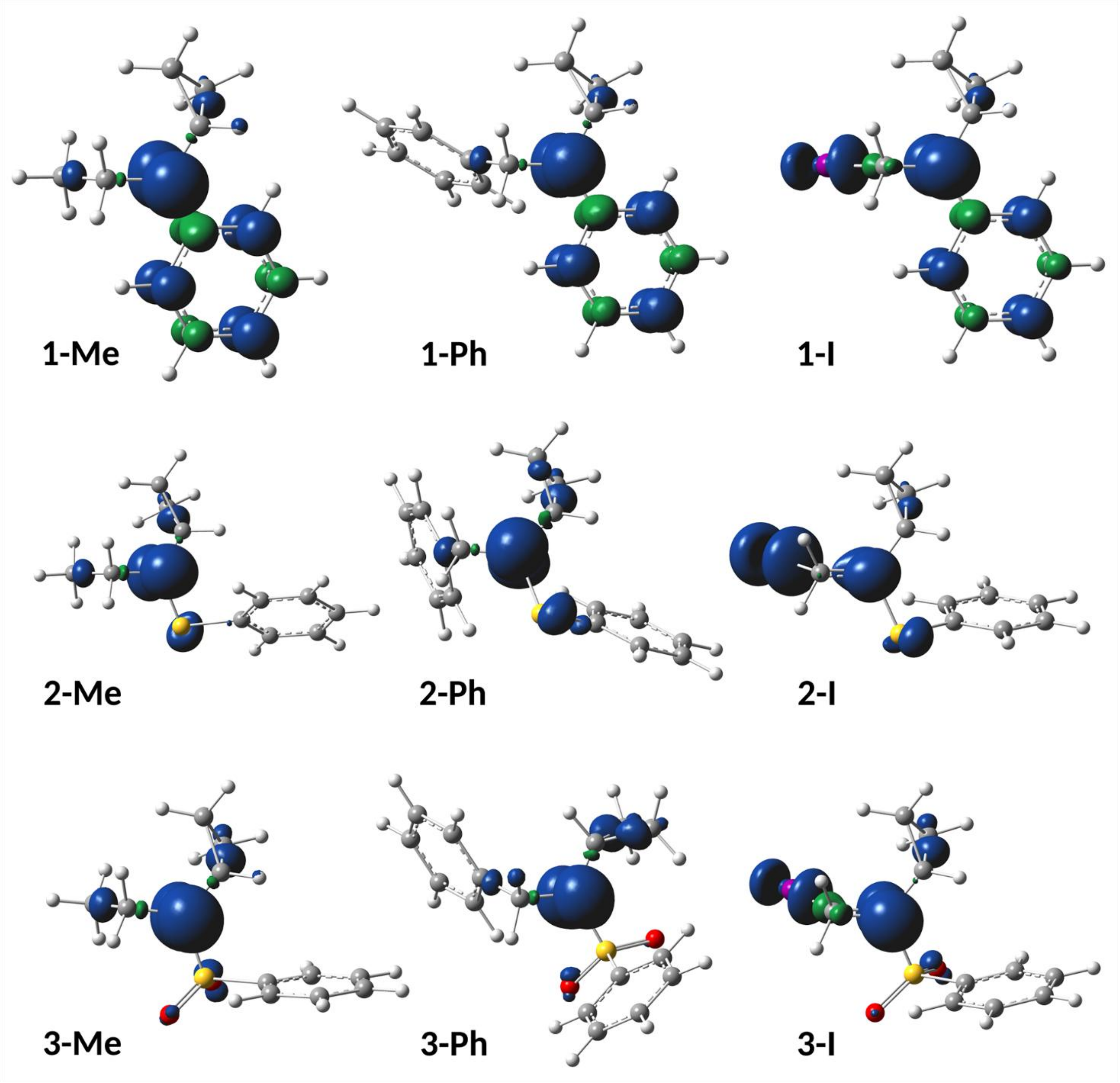

Figure S3. Spatial spin density distribution for the products of radical addition to molecules **1**, **2** and **3**. Products **1-Me**, **2-Me** and **3-Me** correspond to Me-radical addition; products **1-Ph**, **2-Ph** and **3-Ph** correspond to Ph-radical addition; products **1-I**, **2-I** and **3-I** correspond to I-radical addition, UPBE1PBE/6-311+G(d,p) D3BJ.

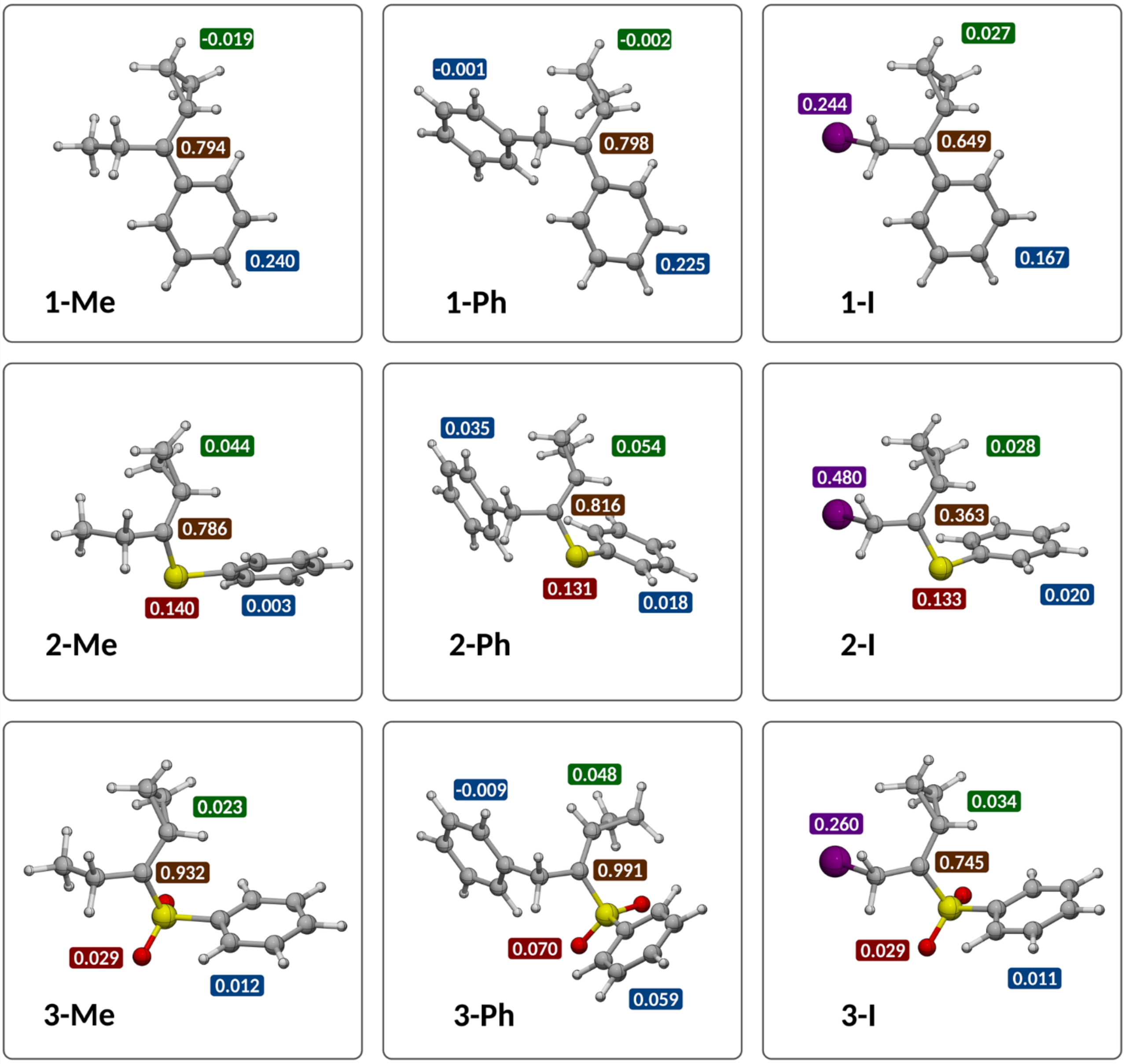

Figure S4. Sum of Mulliken spin populations for different atomic groups: green frame for cyclopropyl substituent, blue frames for phenyl groups, red frame for S atom and SO_2 group, violet frame for iodine atom and brown frame for tertiary radical carbon atom, UPBE1PBE/6-311+G(d,p) D3BJ.

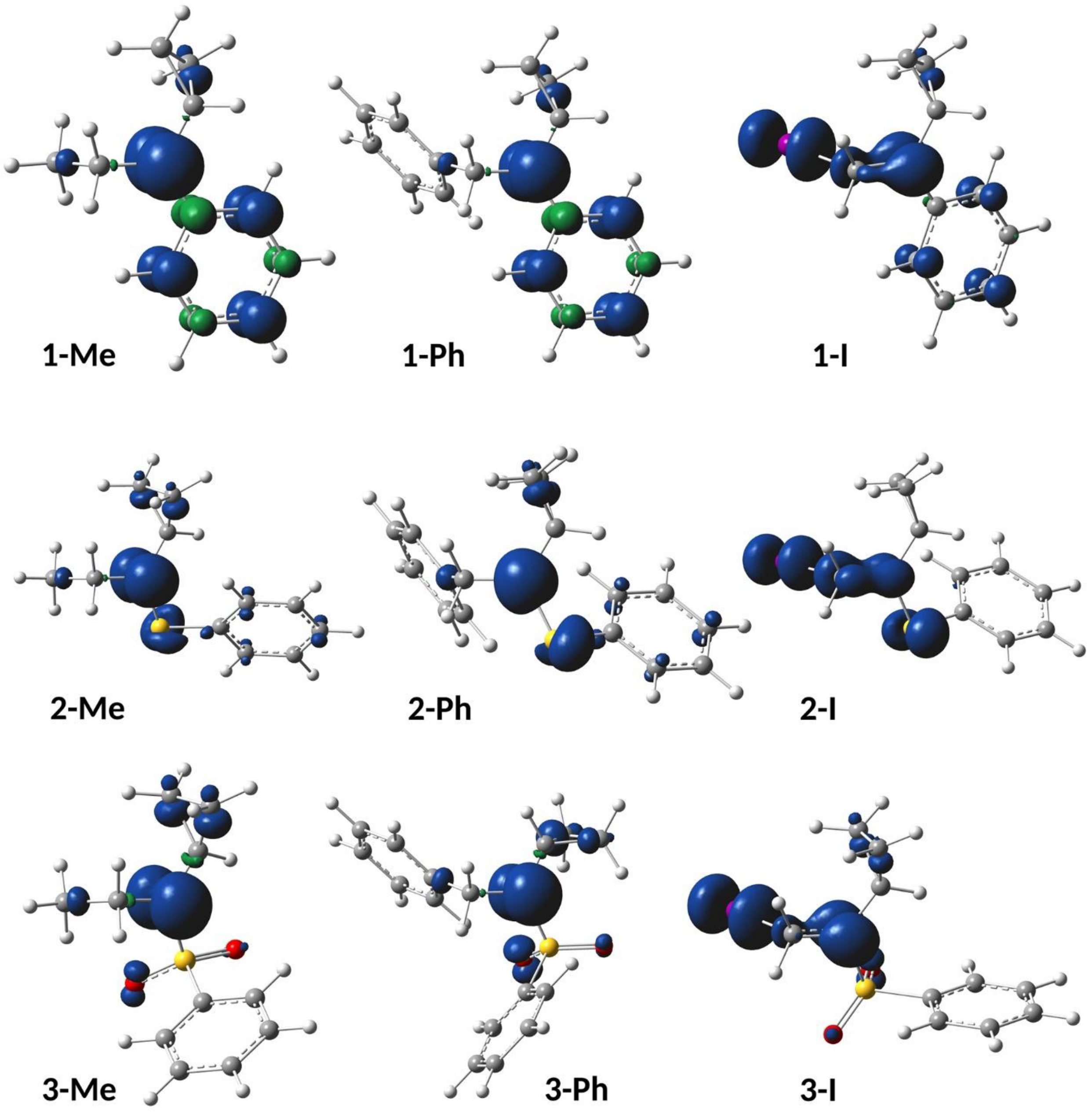

Figure S5. Spatial spin density distribution for the products of radical addition to molecules **1**, **2** and **3**. Products **1-Me**, **2-Me** and **3-Me** correspond to Me-radical addition; products **1-Ph**, **2-Ph** and **3-Ph** correspond to Ph-radical addition; products **1-I**, **2-I** and **3-I** correspond to I-radical addition, UPBE/STO-3G D3.

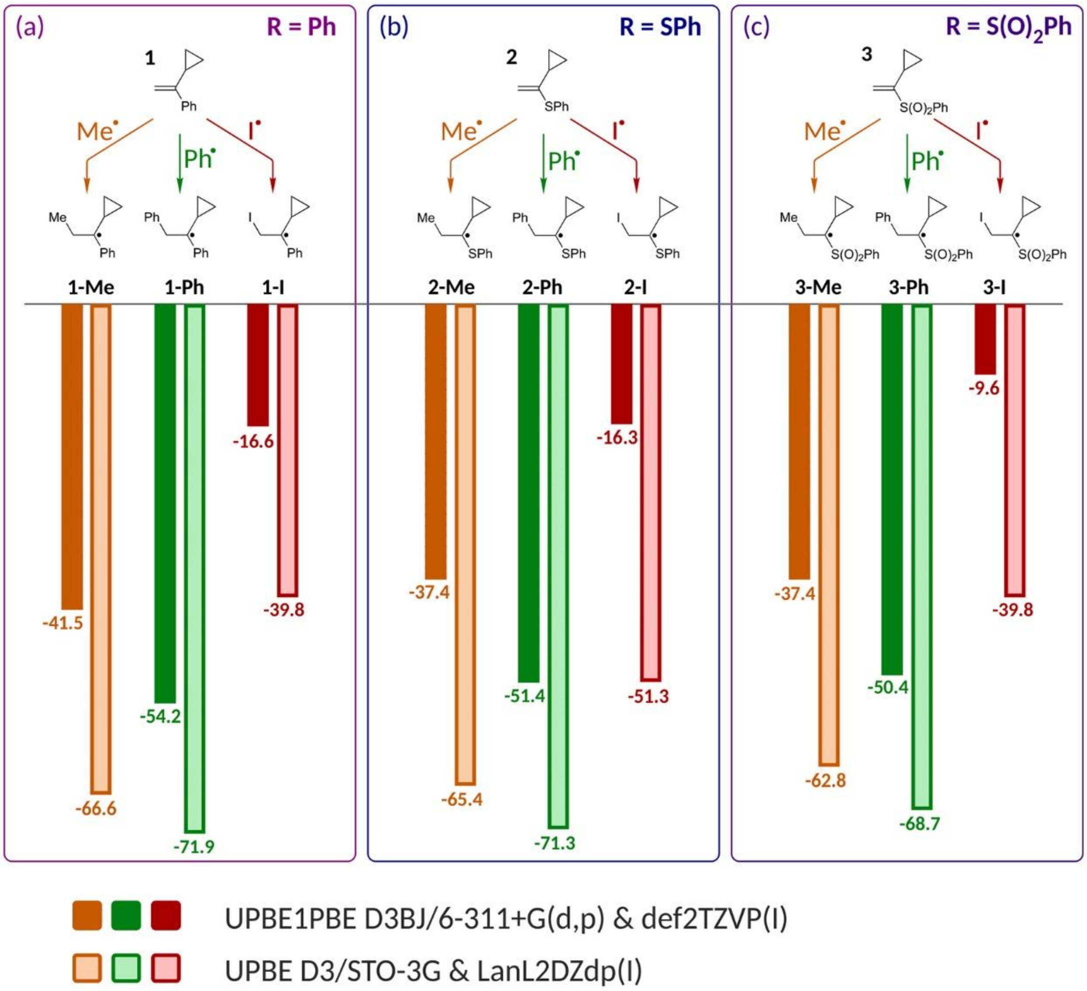

Figure S6. The values of the reaction total energies ΔE (kcal/mol) calculated by different methods for reactions of compounds **1-3** with different radicals.

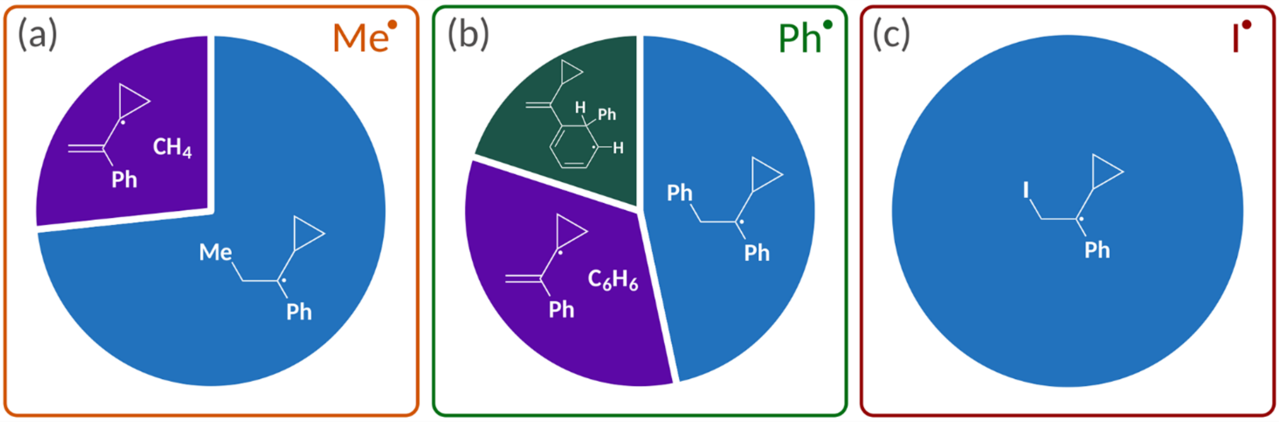

Figure S7. Distribution of products identified as a result of DFT molecular dynamics modelling of radical particle addition to compound **1**: (a) – Me-radical addition; (b) – Ph-radical addition; (c) – I-radical addition.

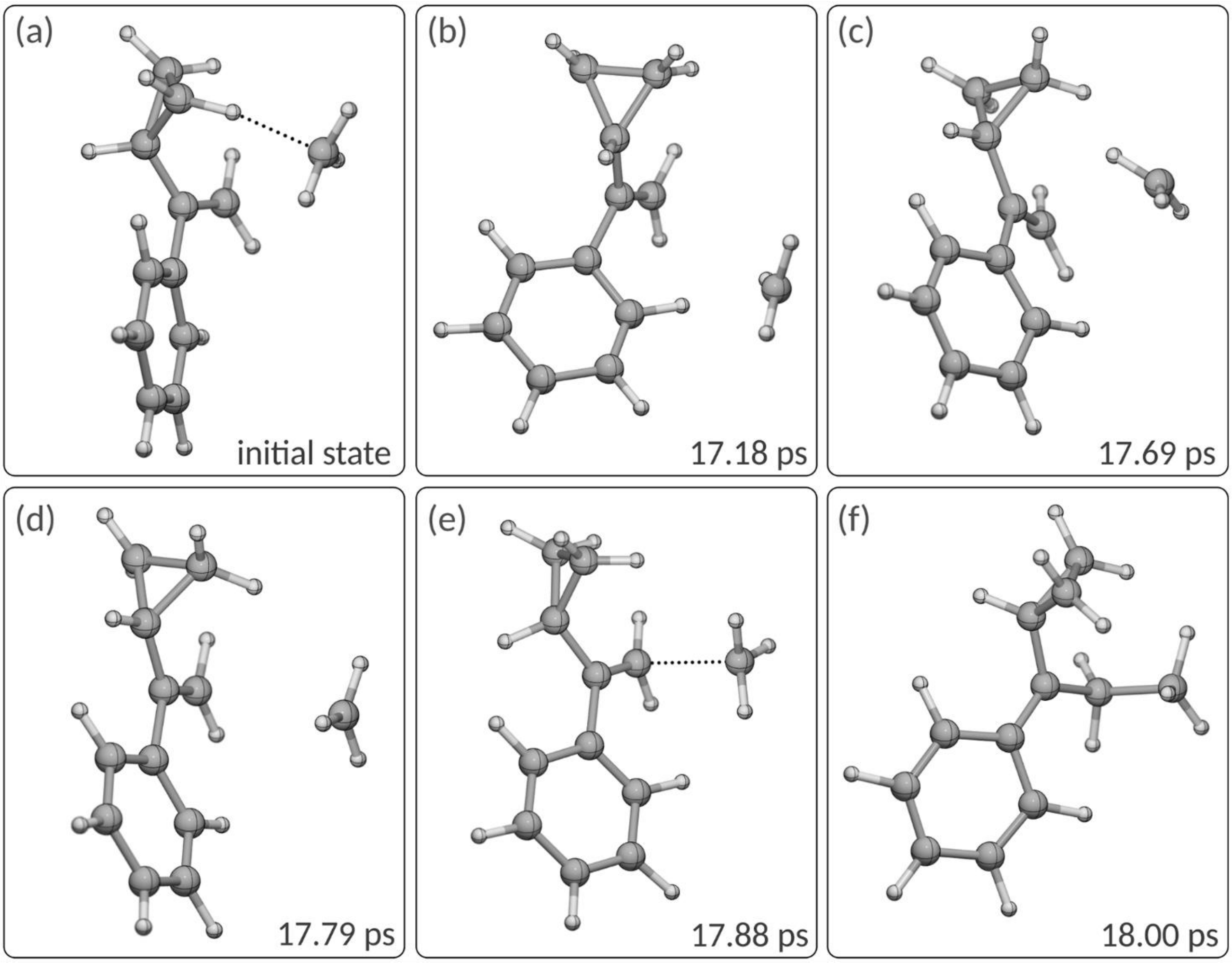

Figure S8. Some frames of one of the MD trajectories resulted in Me-radical addition to the carbon atom of the double bond in compound **1**. (a) – initial state which is optimized molecular structure containing Me-radical coordinated with one of the hydrogen atoms of cyclopropyl substituent; (b, c) – trajectory points showing slight pyramidalization of Me-radical; (d) – immediately before binding, the methyl radical is arranged parallel to the CH_2 group; (e) – molecular system in the process of methyl radical addition; (f) – final state of the methyl radical addition.

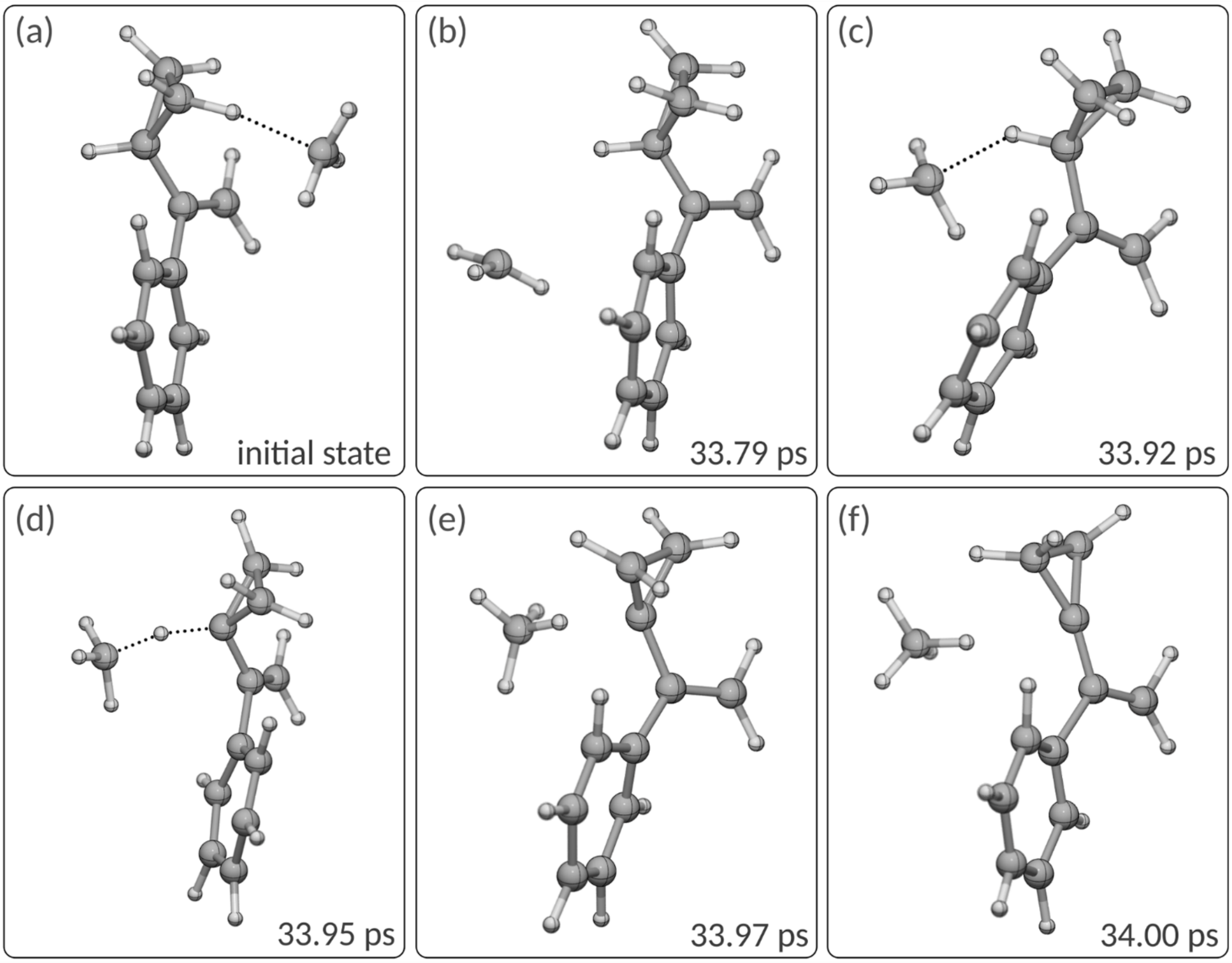

Figure S9. Some frames of one of the MD trajectories end with the formation of a CH_4 molecule as a result of hydrogen atom transfer from compound **1** to Me-radical. (a) – optimized initial state; (b, c) – coordination of Me-radical with hydrogen atom of the cyclopropyl substituent; (d) – hydrogen atom transfer from cyclopropyl group to Me-radical; (e) – complete transfer of the hydrogen atom from cyclopropyl group to Me-radical; (f) – flattening of the tertiary carbon atom of the cyclopropyl substituent and complete detaching of the CH_4 molecule.

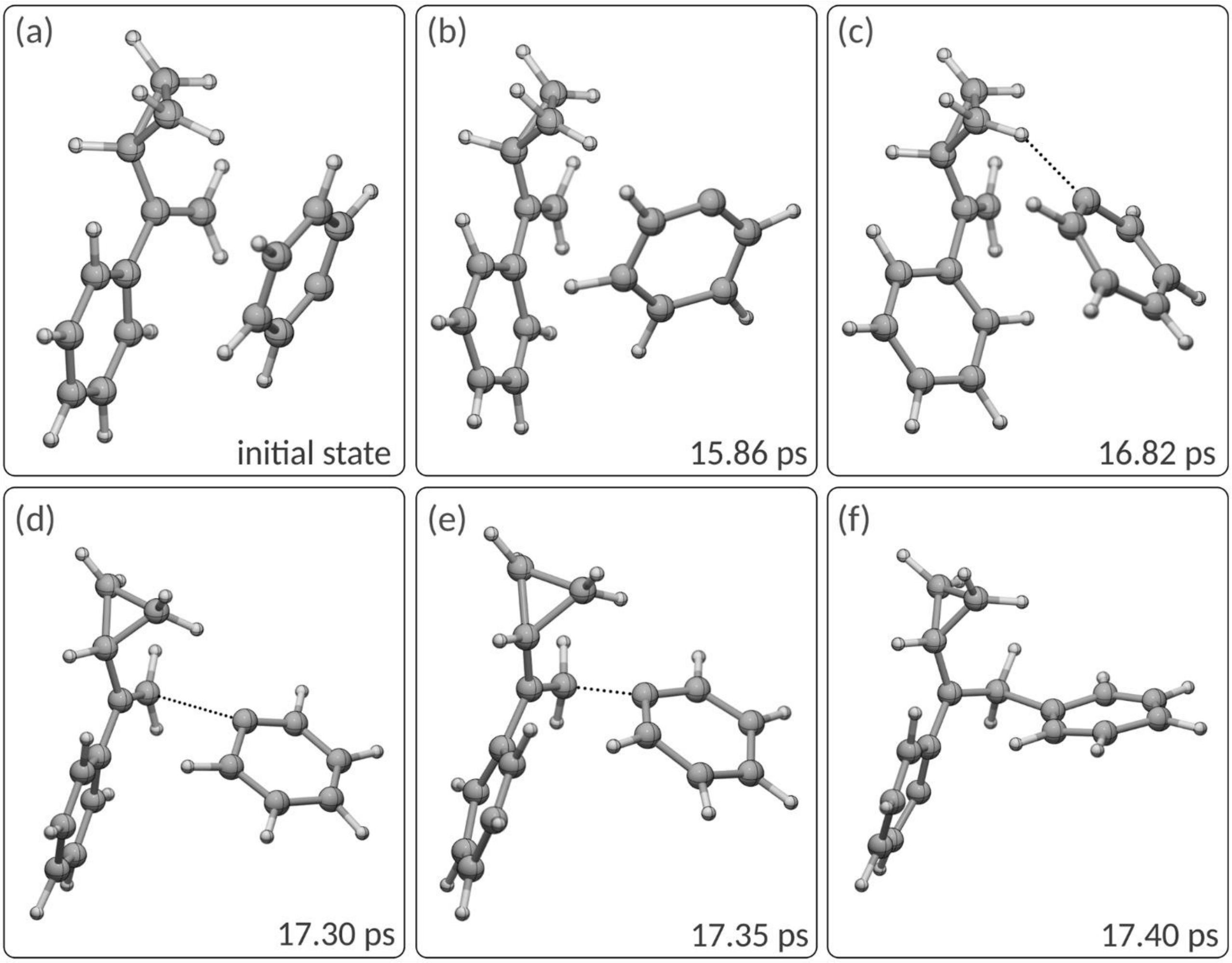

Figure S10. Some frames of one of the MD trajectories resulted in Ph-radical addition to the carbon atom of the double bond in compound **1**. (a) – optimized initial state; (b) – after 15.9 ps, the Ph-radical remains an unbound particle; (c) – weak coordination of the Ph-radical with hydrogen atoms of the cyclopropyl group; (d) – beginning of the Ph-radical binding with terminal carbon atom; (e) – pyramidalization of the terminal carbon atom and C-C bond forming; (f) – final state of the Ph-radical addition.

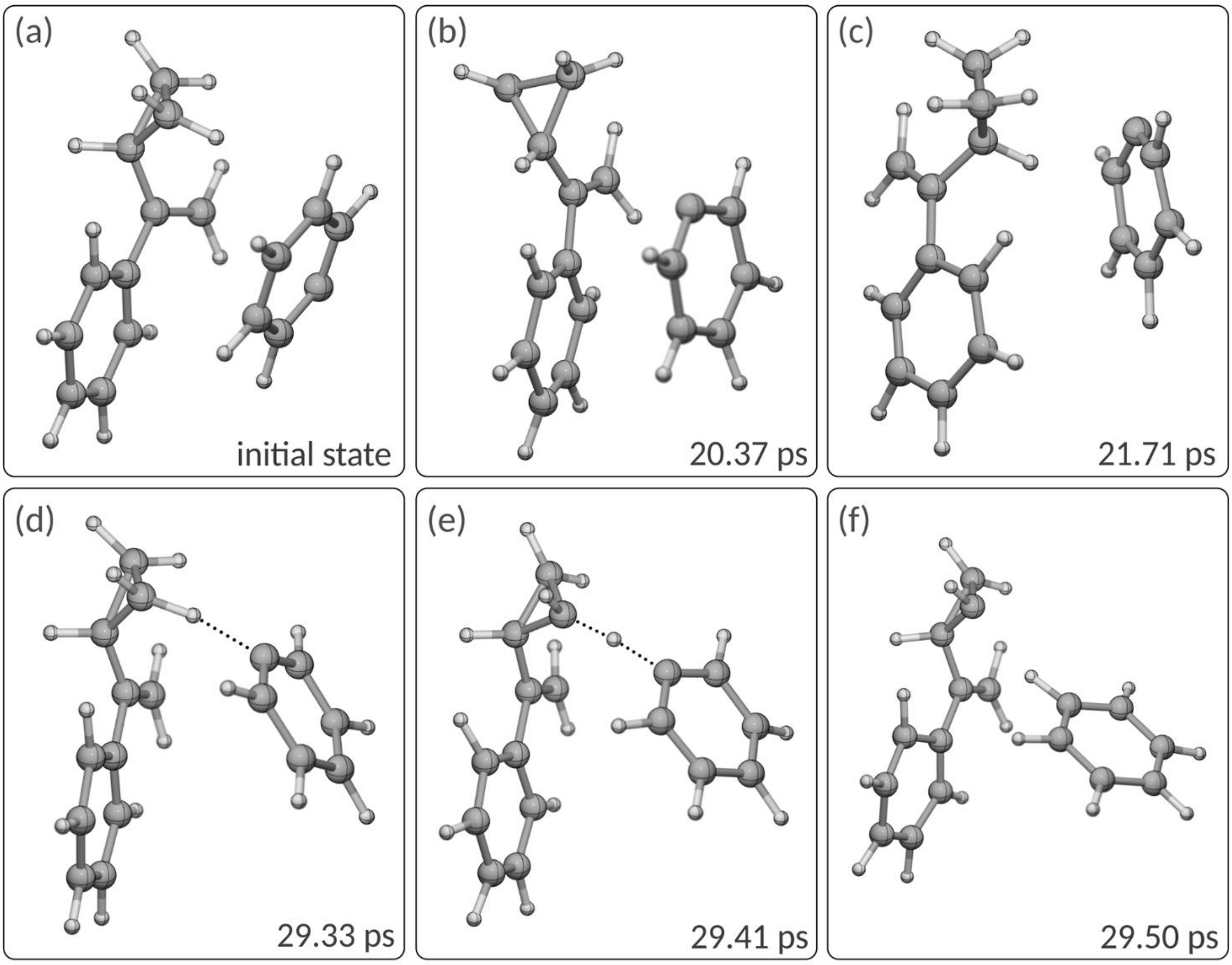

Figure S11. Some frames of one of the MD trajectories end with the formation of the C_6H_6 molecule as a result of hydrogen atom transfer from the secondary carbon atom of compound **1** to the Ph-radical. (a) – optimized initial state; (b) – coordination of Ph-radical with phenyl substituent by stacking; (c) – coordination of Ph-radical with hydrogen atom of cyclopropyl substituent by "plane" of Ph-radical; (d) – coordination of Ph-radical with hydrogen atom of cyclopropyl substituent by radical carbon atom of Ph-radical; (e) – transfer of the hydrogen atom from cyclopropyl group to Ph-radical; (f) – complete transfer of the hydrogen atom from secondary carbon atom of the cyclopropyl substituent to the Ph-radical and formation of the C_6H_6 molecule.

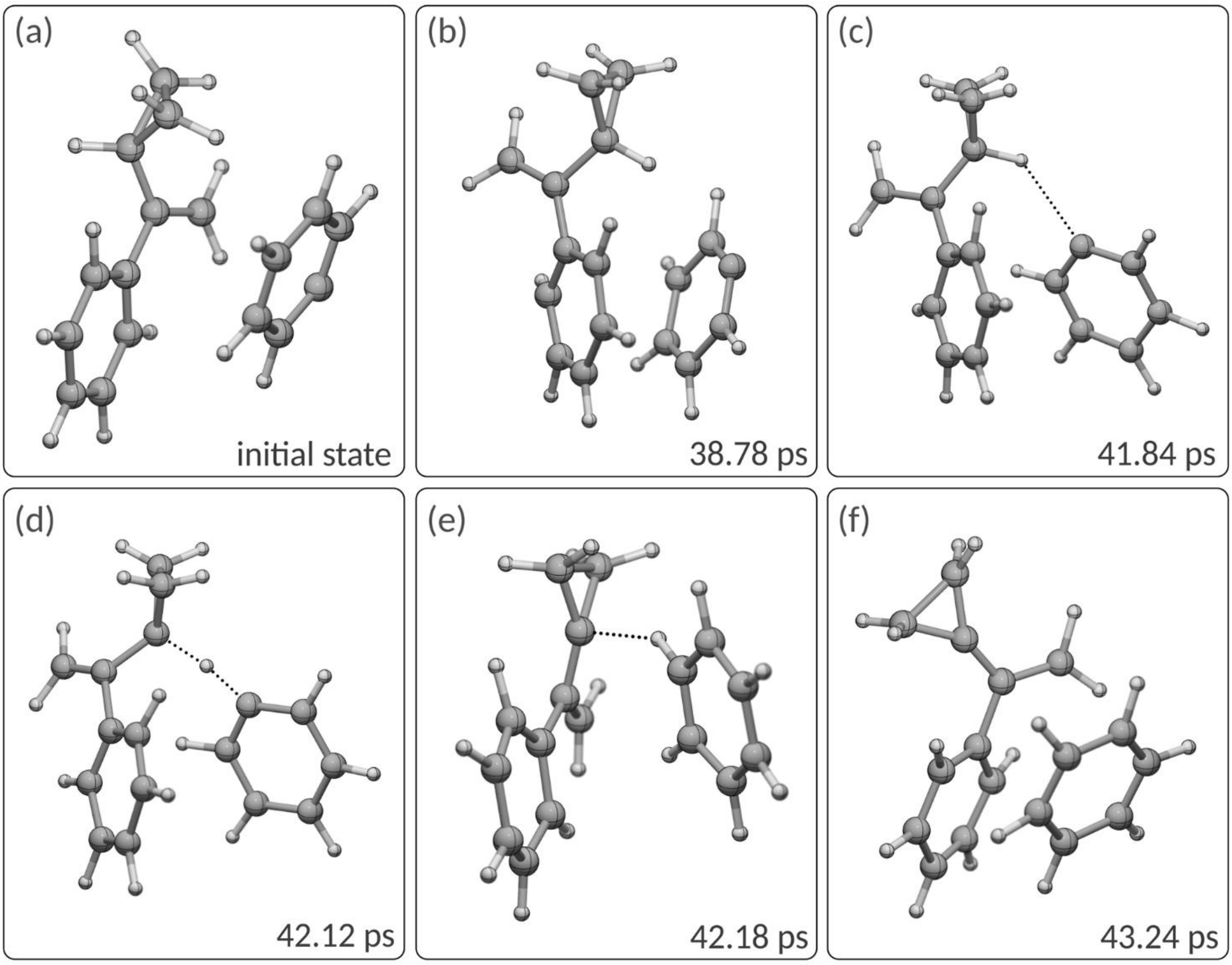

Figure S12. Some frames of one of the MD trajectories ending with the formation of C_6H_6 molecule as a result of hydrogen atom transfer from tertiary carbon atom of compound **1** to Ph-radical: (a) – optimized initial state; (b) – coordination of Ph-radical with phenyl substituent by stacking; (c) – coordination of Ph-radical with hydrogen atom of cyclopropyl substituent by radical carbon atom of Ph-radical; (d) – transfer of the hydrogen atom from tertiary carbon atom of cyclopropyl group to Ph-radical; (e) – complete transfer of the hydrogen atom from tertiary carbon atom of the cyclopropyl substituent to the Ph-radical and formation of the C_6H_6 molecule; (e) – coordination of the benzene molecule with Ph substituent by stacking.

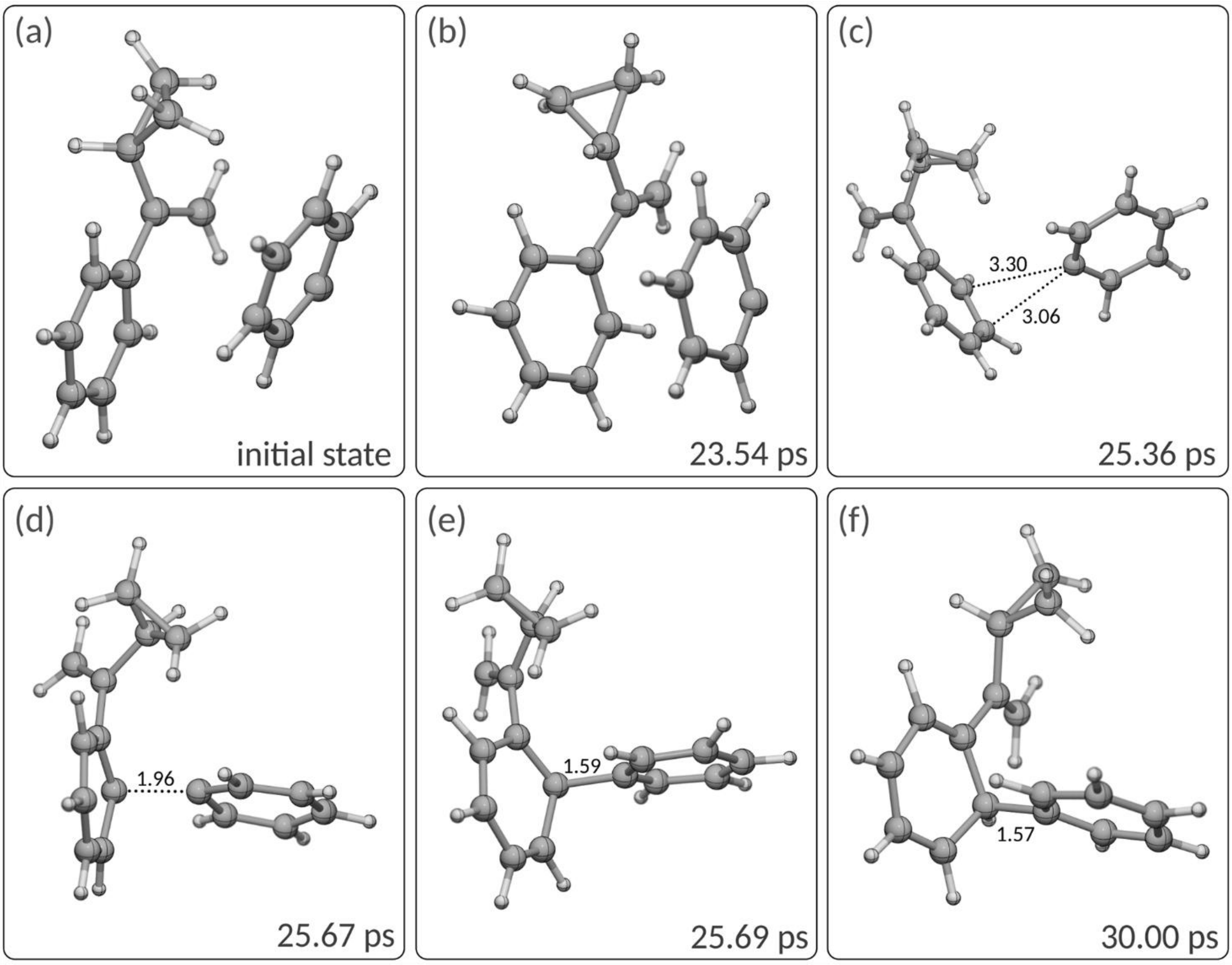

Figure S13. Some frames of one of the MD trajectories ending with the addition Ph-radical to Ph substituent of compound **1** in *ortho*-position: (a) – optimized initial state; (b) – coordination of Ph-radical with phenyl substituent by stacking; (c) – weak coordination of the Ph-radical perpendicularly to the plane of Ph-substituent; (d) – forming of C-C bond between Ph-radical and one of the carbon atoms of the Ph-substituent; (e) – final addition product; (f) – addition product is quite stable during the modelling time.

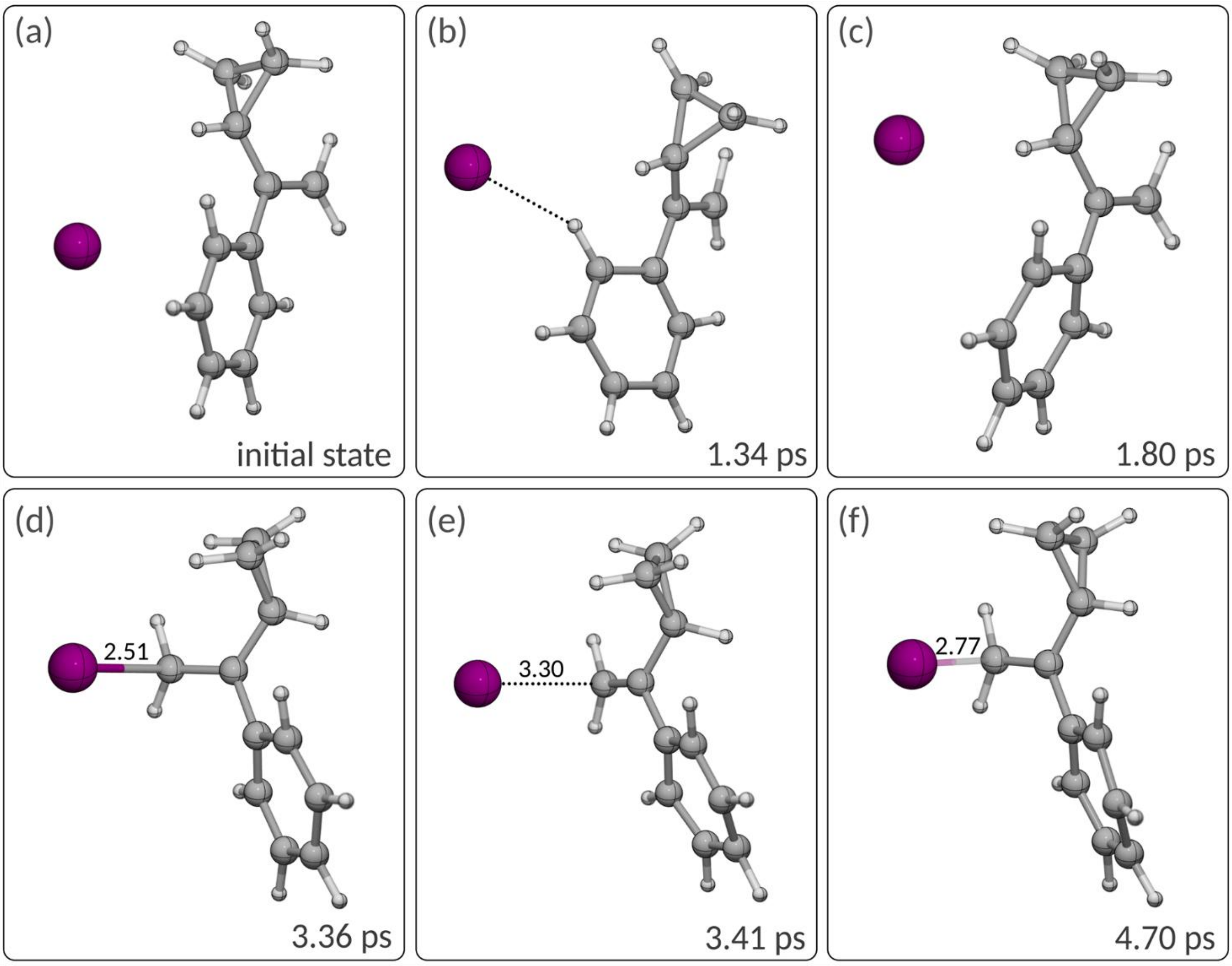

Figure S14. Some frames of one of the MD trajectories ending with the addition I-radical to terminal carbon atom of compound **1**: (a) – optimized initial state; (b, c) – coordination of iodine radical with different atoms without considerable distortion of the molecular structure; (d, e, f) – bonding of iodine radical with terminal carbon atom and C-I bond remain labile.

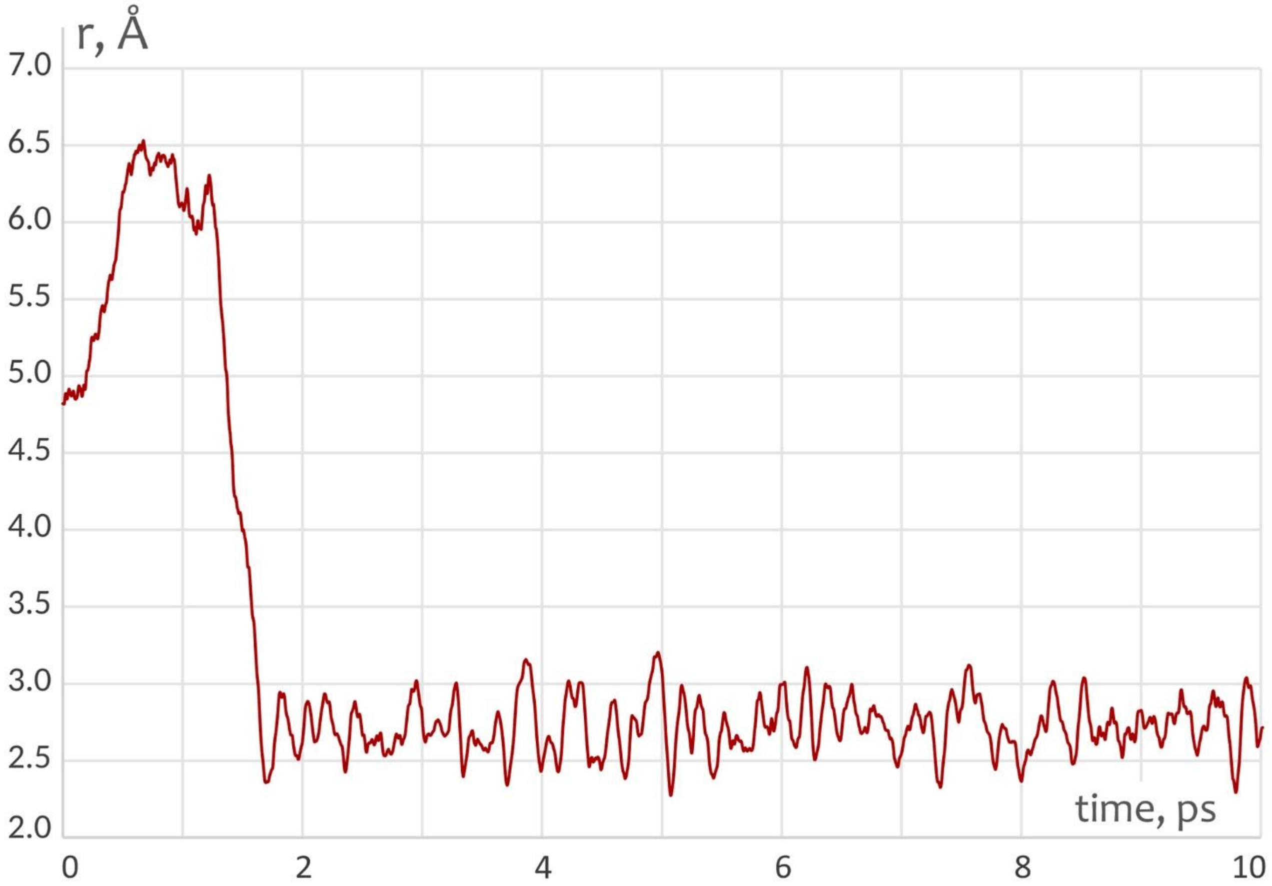

Figure S15. Dynamics of the C-I interatomic distance for the interaction processes of the iodine radical with compound **1**; UPBE/6-31G(d) D3.

4. Experimental study

Synthesis of cyclopropylvinyl derivatives

(1-Cyclopropylvinyl)benzene was prepared according to the published procedure.[12]

(1-Cyclopropylvinyl)(phenyl)sulfide was prepared according to a published procedure.[13]

*Oxidation of Vinyl Sulfide **2** to Vinyl Sulfone **3***

General Procedure:

In an oven-dried 10 mL flask containing a stirring bar, vinyl sulfide **2** (1 mmol) was dissolved in 2 ml CH_2Cl_2. The mixture was cooled in an ice bath, and then MPBCA (2 mmol) was added to the mixture. The reaction was carried out under stirring for 24 h at r.t. After the reaction completion, a $Na_2S_2O_3$ solution was added to the mixture, and the organic layer was separated and washed with $NaHCO_3$ (x2).

((1-cyclopropylvinyl)sulfonyl)benzene (**3**)

Yield 0.3744 g (90%) The product was obtained as yellow liquid.

^{1}H NMR ($CDCl_3$, 300 MHz) ppm: 0.32 – 0.34 (m, 2H), 0.69 – 0.73 (m, 2H), 1.57 – 1.63 (m, 1H), 5.52 (s, 1H), 6.25 (s, 1H), 7.50 – 7.62 (m, 3H), 7.89 – 7.92 (m, 3H).

^{13}C {^{1}H} NMR ($CDCl_3$, 75 MHz) ppm: 7.4, 10.9, 120.2, 128.5, 129.2, 133.5, 139.7, 153.0.

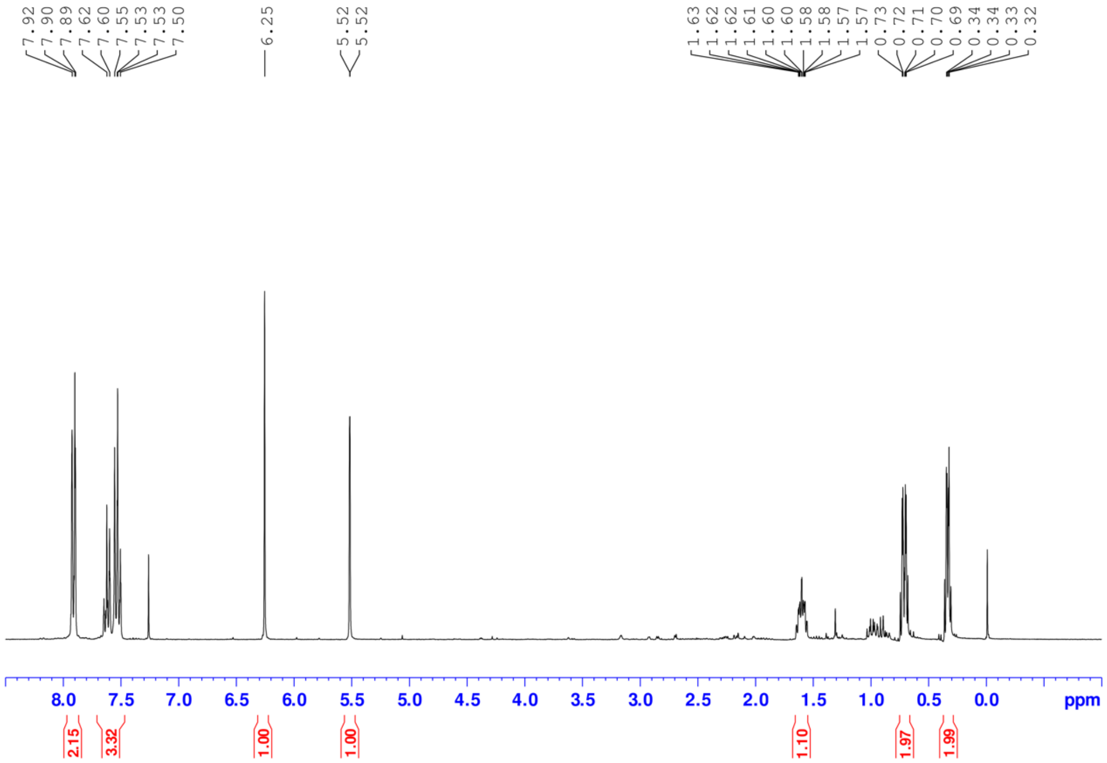

Figure S16. ^{1}H NMR spectrum of **3** in $CDCl_3$.

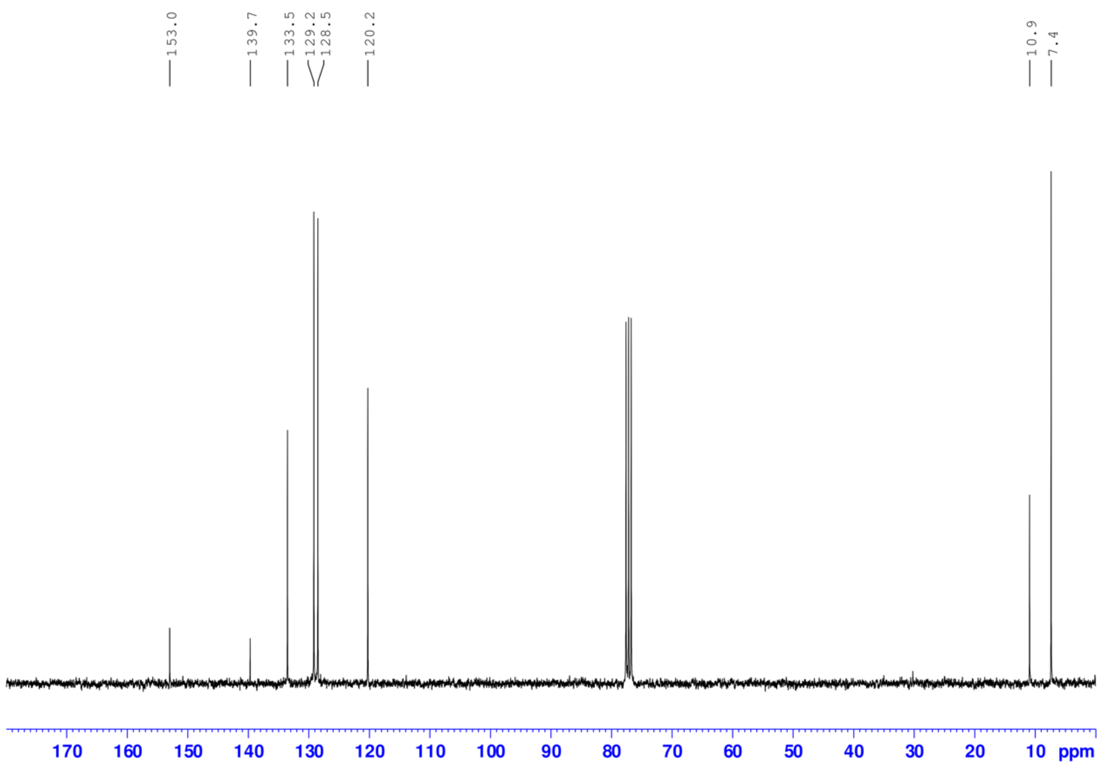

Figure S17. ^{13}C NMR spectrum of **3** in $CDCl_3$.

*Cyclopropane Ring-Opening Reactions of Compounds **1**, **2** and **3** with Alk•*

General Procedure:

An oven-dried 3 mL Wheaton V vial containing a stirring bar was charged with $Pd(OAc)_2$ (0.02 mmol, 10 mol%), xantphos (0.04 mmol, 20 mol%) and Cs_2CO_3 (0.6 mmol, 3 equiv) under a N_2 atmosphere (glovebox). Next, alkyl halide ((iodomethyl)trimethylsilane) (0.3 mmol, 1.5 equiv), alkenes **1**, **2** or **3** (0.2 mmol, 1 equiv) and dry/degassed benzene (1 mL) were added to the reaction vessel via syringes. The vessel was capped with a pressure screw cap and was stirred and heated (100 °C) for 24 h. The resulting mixture was diluted with DCM (5 mL), filtered (Celite), and concentrated under reduced pressure.

This experiment was reproduced according to the published procedure.[14] In the corresponding article, the authors state that reaction between alkyl halide and radical clock resulted in regioselective radical-ring opening of the cyclopropyl unit as a mixture of stereoisomers. Formation of a product of Pd-β-C elimination of the cyclopropane component, or the coupling adduct possessing an intact cyclopropane unit, was not detected.

*Cyclopropane Ring-Opening Reactions of Compounds **1**, **2, 3, 10** and **12** with Ar•*

General Procedure:

To a solution of p-tolyl iodide (0.23 mmol), potassium tert-butoxide (0.69 mmol) and ethanol (0.046 mmol) in DMF (0.50 mL) in a 10 mL Schlenk tube was added compounds **1**, **2**, **3**, **10** or **12** (1.2 mmol). The reaction mixture was stirred at 80 °C for 2 h. After cooling down, the reaction mixture was quenched by water (10 mL) and extracted with diethyl ether (20 mL x 3). The combined organic layer was dried with sodium sulfate, filtered and concentrated.

In the presence of Ar radical, the reaction mechanism is different. The mechanism of this reaction and role of EtOH is not sufficiently studied. Currently, three possible ways of reaction are suggested described in detail in the corresponding article[15]. It is proposed that two isomers are formed. Base-catalyzed isomerization of A is to give B.

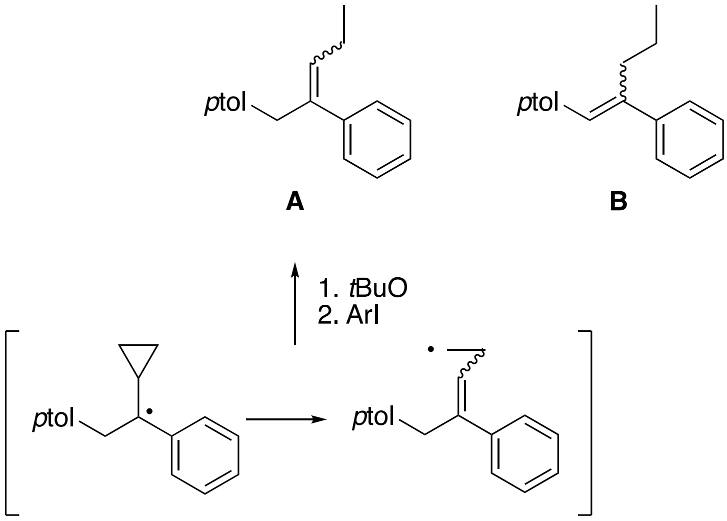

*Investigation of the stability of Compounds **1**, **2** and **3** under UV irradiation with N-iodosuccinimide*

General procedure: containing a stirring bar was dissolved compounds **1**, **2** or **3** (1 mmol) in 2 ml DMSO, and then N-iodosuccinimide (2 mmol) was added to the mixture. The test tube was irradiated under a UV lamp for 1 h.

Decomposition of the initial compound was tracked using NMR. An internal standard (D_2O) was used to determine the amount of decomposed product.

References

[1] a) J. P. Perdew, K. Burke, M. Ernzerhof, *Phys. Rev. Lett.*, 1996, **77**, 3865-3868, DOI: 10.1103/PhysRevLett.77.3865. b) C. Adamo, V. Barone, *J. Chem. Phys.*, 1999, **110**, 6158-6170, DOI: 10.1063/1.478522.

[2] a) A. D. McLean, G. S. Chandler, *J. Chem. Phys.*, 1980, **72**, 5639-5648, DOI: 10.1063/1.438980. b) R. Krishnan, J. S. Binkley, R. Seeger, J. A. Pople, *J. Chem. Phys.*, 1980, **72**, 650-654, DOI: 10.1063/1.438955. c) T. Clark, J. Chandrasekhar, G. W. Spitznagel, P. von R. Schleyer, *J. Comput. Chem.*, 1983, **4**, 294-301, DOI: 10.1002/jcc.540040303.

[3] F. Weigend, R. Ahlrichs, *Phys. Chem. Chem. Phys.*, 2005, **7**, 3297-3305, DOI: 10.1039/b508541a.

[4] a) S. Grimme, J. Antony, S. Ehrlich, H. Krieg, *J. Chem. Phys.*, 2010, **132**, 154104, DOI: 10.1063/1.3382344. b) S. Grimme, S. Ehrlich, L. Goerigk, *J. Comput. Chem.*, 2011, **32**, 1456-1465, DOI: 10.1002/jcc.21759.

[5] Gaussian 16, Revision C.01, M. J. Frisch, G. W. Trucks, H. B. Schlegel, G. E. Scuseria, M. A. Robb, J. R. Cheeseman, G. Scalmani, V. Barone, G. A. Petersson, H. Nakatsuji, X. Li, M. Caricato, A. V. Marenich, J. Bloino, B. G. Janesko, R. Gomperts, B. Mennucci, H. P. Hratchian, J. V. Ortiz, A. F. Izmaylov, J. L. Sonnenberg, D. Williams-Young, F. Ding, F. Lipparini, F. Egidi, J. Goings, B. Peng, A. Petrone, T. Henderson, D. Ranasinghe, V. G. Zakrzewski, J. Gao, N. Rega, G. Zheng, W. Liang, M. Hada, M. Ehara, K. Toyota, R. Fukuda, J. Hasegawa, M. Ishida, T. Nakajima, Y. Honda, O. Kitao, H. Nakai, T. Vreven, K. Throssell, J. A. Montgomery, Jr., J. E. Peralta, F. Ogliaro, M. J. Bearpark, J. J. Heyd, E. N. Brothers, K. N. Kudin, V. N. Staroverov, T. A. Keith, R. Kobayashi, J. Normand, K. Raghavachari, A. P. Rendell, J. C. Burant, S. S. Iyengar, J. Tomasi, M. Cossi, J. M. Millam, M. Klene, C. Adamo, R. Cammi, J. W. Ochterski, R. L. Martin, K. Morokuma, O. Farkas, J. B. Foresman, D. J. Fox, Gaussian, Inc., Wallingford CT, 2016.

[6] CYLview20; C.Y. Legault, Université de Sherbrooke, 2020 (http://www.cylview.org).

[7] GaussView, Version 6, R. Dennington, T. A. Keith, J. M. Millam, Semichem Inc., Shawnee Mission, KS, 2016.

[8] a) W. J. Hehre, R. F. Stewart, J. A. Pople, *J. Chem. Phys.*, 1969, **51**, 2657-2664, DOI: 10.1063/1.1672392. b) J. B. Collins, P. v. R. Schleyer, J. S. Binkley, J. A. Pople, *J. Chem. Phys.*, 1976, **64**, 5142-5151, DOI: 10.1063/1.432189.

[9] a) P. J. Hay, W. R. Wadt, *J. Chem. Phys.*, 1985, **82**, 270-283, DOI: 10.1063/1.448799. b) W. R. Wadt, P. J. Hay, *J. Chem. Phys.*, 1985, **82**, 284-298, DOI: 10.1063/1.448800. c) P. J. Hay, W. R. Wadt, *J. Chem. Phys.*, 1985, **82**, 299-310. DOI: 10.1063/1.448975.

[10] a) I. S. Ufimtsev, T. J. Martinez, *J. Chem. Theory Comput.*, 2008, **4**, 222-231, DOI: 10.1021/ct700268q. b) I. S. Ufimtsev, T. J. Martinez, *J. Chem. Theory Comput.*, 2009, **5**, 1004-1015, DOI: 10.1021/ct800526s. c) I. S. Ufimtsev, T. J. Martinez, *J. Chem. Theory Comput.*, 2009, **5**, 2619-2628, DOI: 10.1021/ct9003004. d) S. Seritan, C. Bannwarth, B. S. Fales, E. G. Hohenstein, C. M. Isborn, S. I. L. Kokkila- Schumacher, X. Li, F. Liu, N. Luehr, J. W. Snyder, C. Song, A. V. Titov, I. S. Ufimtsev, L. P. Wang, T. J. Martínez, *WIREs Comput. Mol. Sci.*, 2020, e1494, DOI: 10.1002/wcms.1494.

[11] W. Humphrey, A. Dalke, K. Schulten, *J. Mol. Graph.*, 1996, **14**, 33-38, DOI: 10.1016/0263-7855(96)00018-5.

[12] M. Gerst, J. Morgenthaler and Ch. Rüchardt, *Chem. Ber.*, 1994, **127**, 691, DOI: 10.1002/cber.19941270419.

[13] L. T. Sahharova, E. G. Gordeev, D. B. Eremin and V. P. Ananikov, *ACS Catal.*, 2020, **10**, 9872, DOI: 10.1021/acscatal.0c02053.

[14] D. Kurandina, M. Parasram, V. Gevorgyan, *Angew. Chem., Int. Ed.*, 2017, **56**, 14212, DOI: 10.1002/anie.201706554.

[15] E. Shirakawa, X. Zhang, T. Hayashi, *Angew. Chem., Int. Ed.*, 2011, **50**, 4671, DOI: 10.1002/ange.201008220.
